# 亚胺连接的多孔共价有机骨架材料结合固相萃取-液相色谱-串联质谱检测蜂蜜中雌激素

**DOI:** 10.3724/SP.J.1123.2022.03017

**Published:** 2022-08-08

**Authors:** Hui LI, Gengbiao REN, Huijuan LI, Xiangfeng CHEN, Zhiguo ZHANG, Yanfang ZHAO

**Affiliations:** 1.齐鲁工业大学(山东省科学院)食品科学与工程学院, 山东 济南 250353; 1. School of Food Science and Engineering, Qilu University of Technology (Shandong Academy of Sciences), Jinan 250353, China; 2.齐鲁工业大学(山东省科学院), 山东省分析测试中心, 山东 济南 250014; 2. Shandong Analysis and Test Centre, Qilu University of Technology (Shandong Academy of Sciences), Jinan 250014, China

**Keywords:** 共价有机骨架材料, 吸附剂, 固相萃取, 液相色谱, 串联质谱, 雌激素, 蜂蜜, covalent organic framework, sorbent, solid-phase extraction (SPE), liquid chromatography (LC), tandem mass spectrometry (MS/MS), estrogen, honey

## Abstract

以亚胺连接的多孔共价有机骨架材料(IL-COF-1)作为固相萃取的吸附剂,建立了液相色谱-串联质谱快速检测蜂蜜样品中痕量雌激素的方法。该研究选择雌二醇、己烯雌酚、雌三醇、*β*-雌二醇和炔雌醇5种雌激素作为目标分析物。在蜂蜜样品中添加雌激素,采用单因素优化法对影响萃取效果的重要因素进行优化,获得最佳条件:IL-COF-1用量为30 mg,样品流速为3 mL/min,样品溶液pH值为7,以5 mL的1%(v/v)氨水-甲醇溶液进行洗脱,流速为0.4 mL/min,萃取过程中不添加NaCl。采用高效液相色谱-三重四极杆质谱联用技术对提取物中的雌激素进行定量分析。以乙腈和5 mmol/L的乙酸铵溶液作为流动相进行梯度洗脱,经C18色谱柱分离,采用电喷雾离子源、质谱多反应监测和负离子扫描模式,实现了蜂蜜样品中5种雌激素的快速定性定量分析。在最佳条件下,方法验证结果中雌三醇、*β*-雌二醇和炔雌醇的线性范围为1~500 ng/g,雌二醇和己烯雌酚的线性范围为0.1~100 ng/g,相关系数(*r*)为0.9934~0.9972。检出限(*S/N*=3)为0.01~0.30 ng/g,定量限(*S/N*=10)为0.05~0.95 ng/g。添加50 ng/g 5种雌激素进行重复性实验,日内精密度相对标准偏差(RSD)为3.2%~6.6%,日间精密度RSD为4.2%~7.9%。基于IL-COF-1的固相萃取-液相色谱-串联质谱法具有快速准确、灵敏度高等特点,适用于蜂蜜中雌激素的分析和检测。将该方法应用于4个实际蜂蜜样品中雌激素的检测,均未检出目标物;在低中高3个水平下,5种雌激素的加标回收率为80.1%~115.2%,结果令人满意。

## Abstract (en)

This study aimed to establish a method for the rapid determination of trace estrogens in honey samples by high performance liquid chromatography-tandem mass spectrometry (HPLC-MS/MS) using imine-linked porous covalent organic framework material (IL-COF-1) as the adsorbent for solid-phase extraction (SPE). Estradiol (E_1_), diethylstilbestrol (DES), estriol (E_3_), *β*-estradiol (E_2_), and ethinylestradiol (EE_2_) were used as the target analytes. A single factor optimization method was performed to optimize the extraction effect by adding estrogens to honey samples. The optimal conditions were as follows. A total of 30 mg IL-COF-1 was filled in the SPE column. The sample pH was adjusted to 7. The sample was loaded at a flow rate of 3 mL/min and eluted with 5 mL of a 1% (v/v) NH_3_·H_2_O-methanol solution. The flow rate of the eluent was 0.4 mL/min. NaCl was not added in the extraction process. HPLC coupled to electrospray ionization and triple quadrupole mass spectrometry was introduced to quantify the estrogens in the extracts. The estrogens were separated on a Thermo Fisher Scientific C18 analytical column (100 mm×2.1 mm, 5 μm). Acetonitrile and 5 mmol/L ammonium acetate solution were used as the mobile phases for gradient elution. The column temperature was set at 40 ℃, and the autosampler temperature was maintained at 10 ℃. The rapid qualitative and quantitative analysis of the five estrogens in the honey samples was operated under multiple reaction monitoring mode in a negative electrospray ion source mode. IL-COF-1 prepared in six batches was used as a filler for the SPE column. The relative standard deviations (RSDs) of the recoveries of the estrogens among different batches were 5.2%-9.1%. The reusability of IL-COF-1 material was assessed. After six SPE cycles on the same solid-phase extraction column, the RSDs of the estrogen recoveries were 2.5%-6.1%, indicating that IL-COF-1 has good reusability. The recoveries of estrogens obtained on an IL-COF-1 solid-phase extraction column within 6 days (tested once a day) were 95.1%-107.4%, and the RSDs were 6.2%-8.9%. These results confirmed that the SPE filler had good stability. The method validation results showed that the linear detection ranges were 1-500 ng/g for E_3_, E_2_, and EE_2_, and 0.1-100 ng/g for E_1_ and DES withe the correlation coefficients of 0.9934-0.9972. The limits of detection (LODs, *S/N*=3) were 0.01-0.30 ng/g, and the limits of quantification (LOQs, *S/N*=10) were 0.05-0.95 ng/g. Five estrogens were added (50 ng/g) for the repeated experiments. The RSDs of the intra-day precision were 3.2%-6.6%. The RSDs of the inter-day precision were 4.2%-7.9%. This method was applied to determine the estrogen levels in four honey samples, and no estrogen was found. The recoveries of the five estrogens in sample spiked at three levels including low, middle, and high levels were investigated, and satisfactory recoveries (80.1%-115.2%) were obtained. The SPE-HPLC-MS/MS method based on IL-COF-1 is rapid, accurate, and sensitive, making it suitable for analyzing and detecting estrogen in honey. Further exploration of the use of IL-COF-1 for the extraction processes is in progress.

雌激素是一种重要的内分泌干扰化合物^[1⇓-3]^,与人类身体器官的癌变有很大的关系,如乳腺癌、前列腺癌和卵巢癌等^[4⇓⇓-7]^。近年来,在环境水体、表层沉积物和牛奶中均检测到了雌激素^[8⇓⇓-11]^,蜂蜜中的雌激素可能来自于环境污染和人工添加^[12,13]^。根据美国食品药品监督管理局(Food and Drug Administration, FDA)和欧洲食品安全管理局(European Food Safety Authority, EFSA)的规定,蜂蜜中禁止添加雌激素^[14]^。蜂蜜中的雌激素含量通常较低,检测时会伴有许多干扰物质。但是目前国内并没有相关的标准检测方法,所以需要发展一种简单、灵敏、快速的检测方法,以应对蜂蜜样品中痕量雌激素的检测需求。

目前雌激素常用的分析方法有液相色谱-质谱法(LC-MS)^[15⇓⇓-18]^和气相色谱-质谱法(GC-MS)^[19⇓-21]^。样品在色谱或质谱分析之前,需要富集目标物。各种预处理方法中,固相萃取(SPE)技术具有重现性好、回收率高、萃取时间短、有机溶剂消耗量低等优点,被认为是雌激素样品预处理的有效技术^[22]^。利用聚苯乙烯-二乙烯基苯(HLB)吸附剂萃取蜂蜜中雌激素时,其他杂质成分可能会吸附在HLB吸附剂上,干扰雌激素的富集^[18]^。这些干扰物的共吸附不仅影响雌激素的保留,还会产生基质效应。因此,通常需要前处理程序来减少基质效应,提高方法精度。共价有机骨架材料(covalent organic framework materials, COF)是一种新型多孔材料,由轻质元素组成,通过强共价键与有机单体连接^[22,23]^。因为COF具有大的负载容量、可调节的孔径、高比表面积、高热稳定性和化学稳定性^[24,25]^等优点,所以被广泛应用于固相萃取技术。

在本研究中,我们制备了以亚胺连接的多孔共价有机骨架材料(imine-linked porous covalent organic framework material, IL-COF-1),作为SPE的吸附剂,用来富集蜂蜜中的雌激素。采用单因素优化法对固相萃取条件进行优化,提出了一种用于蜂蜜中雌激素定量检测的固相萃取-高效液相色谱-串联质谱(SPE-HPLC-MS/MS)方法。

## 1 实验部分

### 1.1 仪器、试剂与材料

SWPRA^TM^55扫描电子显微镜(德国蔡司公司), Nicolet710傅里叶变换红外光谱仪(美国尼高力公司), Empyrean锐影X射线衍射仪(XRD, 荷兰PANAlytical B. V.公司), ASAP 2020型比表面积测定仪(美国Micromeritics公司), JEM-7500型透射电子显微镜(TEM),高速离心机(Heraeus Multifuge X1R,美国赛默飞公司),液相色谱-三重四极杆质谱仪(AB SCIEX Triple Quad TM 5500,美国AB SCIEX公司)。

1,3,6,8-四(4-甲苯基)芘(TFPPy)、对苯二胺(*p*-PDA)、1,2-二氯苯、1-丁醇、乙酸购于国药集团化学试剂有限公司(中国上海);雌酮(E_1_)、己烯雌酚(DES)、雌三醇(E_3_)、*β*-雌二醇(E_2_)、炔雌醇(EE_2_)购于TCI (日本东京);甲醇、乙腈、丙酮均为HPLC级,购于默克有限公司(德国);空柱管、筛板购于Biocomma Limited (中国深圳);实验用水为超纯水。

### 1.2 样品制备及溶液配制

#### 1.2.1 样品制备

蜂蜜样品购于当地零售市场,首先用20 mL超纯水稀释1 g蜂蜜于离心管内,然后涡旋2 min。使用0.45 μm微孔滤膜(水系,尼龙)过滤,备用。

#### 1.2.2 溶液配制

单标准储备溶液:称取适量雌激素标准品,用甲醇溶解,配成1000 mg/L标准储备溶液;混合标准溶液:分别准确移取适量单标准储备溶液,以甲醇定容,配制成10 mg/L的标准混合溶液。4 ℃保存,使用时稀释成所需浓度。

### 1.3 材料及固相萃取装置的制备

#### 1.3.1 材料制备

IL-COF-1样品是依据文献^[25]^方法合成的。准确称取TFPPy (80 mg, 0.128 mmol)与*p*-PDA (28 mg, 0.256 mmol)固体粉末置于Pyrex管中,然后加入1,2-二氯苯(2 mL)和1-丁醇(2.0 mL)。在室温条件下超声处理1 min,向溶液中添加0.4 mL的醋酸水溶液(6 mol/L)。随后,将试管放入液氮浴中,快速冷冻、抽真空解冻,反复3次。然后将混合溶液升温至120 ℃反应72 h,自然冷却至室温,通过离心分离固体反应产物,用甲醇洗涤,在60 ℃下真空干燥12 h,得到浅黄色固体粉末,即为IL-COF-1。IL-COF-1的元素分析结果:C, 87.39%; N, 7.84%; H, 4.76%。

#### 1.3.2 固相萃取装置的制备

将30 mg IL-COF-1填入已放入垫片的SPE空柱管(体积3 mL)中,铺平,将另一片垫片轻压在材料上,组装SPE柱。将组装好的SPE柱放置在固相萃取装置上,装置的一端与真空泵连接,另一端与采样器连接,采样器的末端插入水样中,用于抽取实际稀释的蜂蜜样品。

使用前用2 mL甲醇、超纯水对SPE柱进行活化;然后上样,20 mL实际蜂蜜样品(调节溶液pH=7)以3 mL/min的速度通过SPE柱;上样后用5 mL超纯水淋洗吸附在SPE柱上的杂质;常压干燥5 min,用5 mL 1%(v/v)氨水-甲醇溶液洗脱。洗脱液氮吹至干,残渣用1 mL甲醇溶解,涡旋混匀30 s后,过0.22 μm微孔滤膜(有机系统,尼龙),用HPLC-MS/MS分析。

### 1.4 HPLC-MS/MS条件

色谱条件 Thermo Fisher Scientific C18色谱柱(150 mm×2.1 mm, 5 μm);流动相A为乙腈,B为5 mmol/L的乙酸铵;等度洗脱:0~5 min, 45%A和55%B。柱温40 ℃,自动进样器温度10 ℃。

质谱条件 电离方式:ESI^-^;扫描方式:多反应监测(MRM)模式;气帘气压力:275 kPa (40 psi);喷雾电压:5.5 kV;离子源温度:500 ℃;雾化气压力:345 kPa (50 psi);碰撞气、雾化气:N_2_。外标法定量,其他参数见表1。

**表1:** 雌激素的多反应监测模式参数

Compound	Formula	t_R_/min	Precursor ion (m/z)	Fragment ion (m/z)	Clustering voltage/V	Collision energy/V
E_3_	C_18_H_24_O_3_	0.90	286.9	144.8	-110	-47
				170.9	-110	-45
E_2_	C_18_H_24_O_2_	1.68	270.9	145.0	-100	-57
				182.8	-100	-45
EE_2_	C_20_H_24_O_2_	2.02	295.0	144.8	-110	-50
				269.0	-110	-50
E_1_	C_18_H_24_O_2_	2.25	266.8	250.9	-100	-40
				236.8	-100	-40
DES	C_18_H_20_O_2_	2.60	272.8	185.0	-100	-57
				147.0	-100	-50

E_3_: estriol; E_2_: *β*-estradiol; EE_2_: ethinylestradiol; E_1_: estradiol; DES: diethylstilbestrol.

## 2 结果和讨论

### 2.1 IL-COF-1的表征

如图1a所示,扫描电子显微镜图像显示IL-COF-1呈均匀的珊瑚状形态,具有约30~70 nm的聚集立方体形貌,与透射电子显微镜图像显示的IL-COF-1形态一致(见图1b)。XRD图谱显示了IL-COF-1的晶体结构,观察到5个衍射峰分别在(110)、(020)、(220)、(130)和(330)处,与文献^[25]^数据一致(见图1c)。IL-COF-1的BET比表面积为2023 m^2^/g(见图1d)。孔径约为3 nm (见图1e)。傅里叶红外光谱(FT-IR)如图1f所示,1621 cm^-1^处的峰显示特征C=N键。光谱中还观察到C=O (1623 cm^-1^)和N-H (1603 cm^-1^)振动,这验证了IL-COF-1表面存在醛和胺官能团化的单体,有利于雌激素和IL-COF-1之间发生分子间O-H…N=C氢键相互作用。

**图1:**
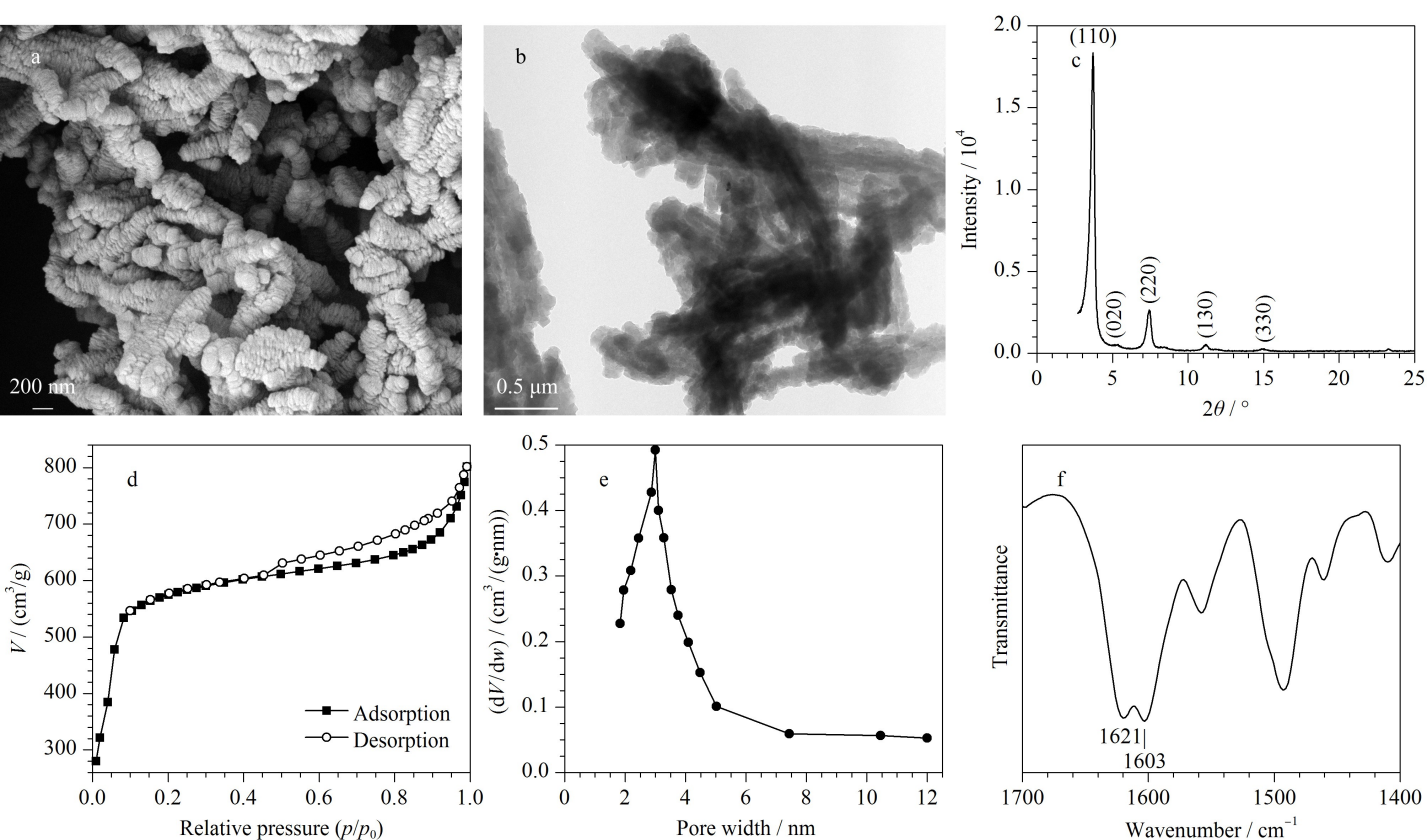
IL-COF-1材料的表征

### 2.2 固相萃取条件的优化

对IL-COF-1吸附剂的吸附能力进行验证。以E_1_、E_2_、E_3_、EE_2_和EDS 5种雌激素作为目标物,E_3_、E_2_和EE_2_的含量为10 ng/g, E_1_和DES的含量为1.0 ng/g,利用这些化合物的回收率评价IL-COF-1对雌激素的萃取效果。为了获取最优的固相萃取条件,优化了IL-COF-1的用量、样品溶液pH、样品流速、洗脱液体积、洗脱液流速、样品溶液中NaCl的含量及洗脱液类型。

考察了20~40 mg IL-COF-1用量对样品回收率的影响(见图2a)。结果表明,使用30 mg吸附剂的回收率最高。因此,实验中使用30 mg IL-COF-1作为固相萃取填料。IL-COF-1的用量与其高比表面积密切相关。

**图2:**
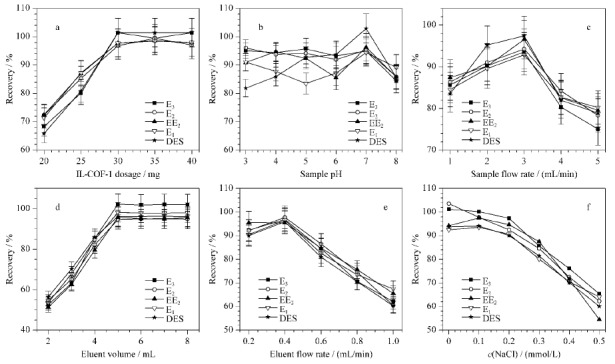
(a) IL-COF-1的量、(b)样品pH、(c)样品流速、(d)洗脱液体积、(e)洗脱液流速和(f) NaCl浓度对雌激素萃取效率的影响(*n*=6)

样品pH值是影响雌激素回收率的重要因素之一,与样品溶液中目标物的存在状态有关。选取样品pH值范围为3~8,如图2b所示,在pH=7时,5种雌激素的回收率最高。可能是因为雌激素和IL-COF-1之间的相互作用主要有*π-π*相互作用和疏水相互作用。IL-COF-1与目标物之间的作用力与目标物的状态有关,不同的状态可能产生不同的作用力。在pH=7时,化合物以分子状态为主,IL-COF-1与目标物之间的作用力最强。因此,选择pH=7。

样品流速是影响目标物质滞留时间和预处理时间的主要因素,考察了样品流速在1~5 mL/min 范围内的萃取效果。在1~3 mL/min范围内,随着样品流速增大,回收率增大,一直保持在80%以上(见图2c)。3~5 mL/min范围内,回收率随样品流速增加而下降。因此,采用3 mL/min作为适宜的样品流速。

考察了洗脱液(1%(v/v)氨水-甲醇溶液)体积在2~8 mL范围内的萃取效果(见图2d)。在2~5 mL范围内,随着洗脱液体积的增加,雌激素回收率逐渐升高。而洗脱液超过5 mL后回收率几乎保持不变(见图2d)。所以,实验中洗脱液体积选择5 mL。

洗脱液流速显著影响雌激素和IL-COF-1之间的接触反应时间。考察了洗脱液(1%(v/v)氨水-甲醇溶液)流速在0.2~1.0 mL/min范围内的萃取效果。在0.2、0.4 mL/min时,回收率维持在85%以上(见图2e)。0.4~1.0 mL/min范围内,回收率随洗脱液流速增加而下降。因此,选择0.4 mL/min作为最佳洗脱液流速。

考察了NaCl对目标物质萃取效率的影响。将NaCl添加到20 mL的样品溶液中,配制含不同浓度NaCl样品溶液进行萃取实验。如图2f所示,当NaCl浓度在0~0.2 mmol/L范围时,回收率出现缓慢下降的趋势。0.2~0.5 mmol/L范围时回收率下降速度增加。所以,在萃取过程中不添加NaCl。

在SPE过程中,洗脱液种类是影响萃取效率的关键因素。考察了不同类型的洗脱液,包括甲醇、乙腈、乙酸乙酯、1%(v/v)氨水-甲醇溶液、1%(v/v)氨水-乙腈溶液和1%(v/v)氨水-乙酸乙酯溶液对萃取效果的影响(见图3)。当使用1%(v/v)氨水-甲醇溶液作为洗脱液时,5种目标物质的回收率最高。因此,选择1%(v/v)氨水-甲醇溶液作为本实验的洗脱液。

**图3:**
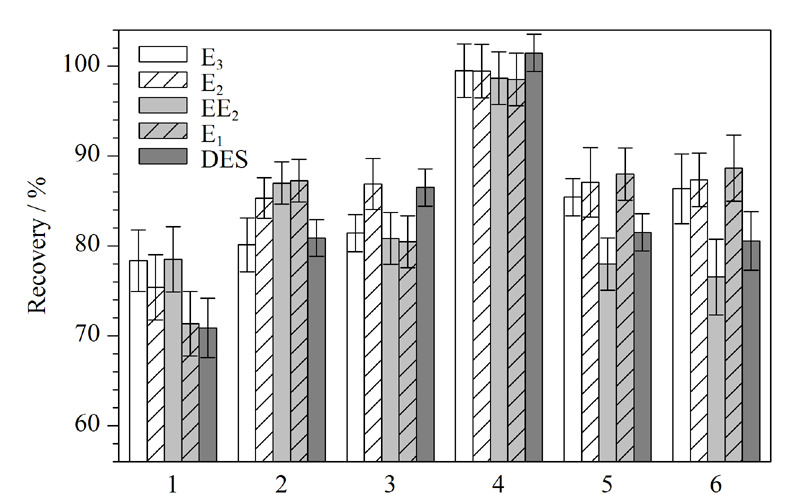
洗脱液种类对雌激素萃取效率的影响(*n*=6)

综上,5种雌激素的最佳萃取条件为:IL-COF-1用量为30 mg, 1%(v/v)氨水-甲醇溶液作为洗脱液,洗脱液体积为5 mL,洗脱液流速为0.4 mL/min,样品流速为3 mL/min,样品pH=7,萃取过程中不添加NaCl。

### 2.3 IL-COF-1的稳定性和重复性

以E_1_、E_2_、E_3_、EE_2_和EDS 5种雌激素作为目标物,E_3_、E_2_和EE_2_的含量为10 ng/g, E_1_和DES的含量为1.0 ng/g。利用回收率评价IL-COF-1的重复性和稳定性,采用最佳萃取条件进行实验。

IL-COF-1的制备重复性 以6个批次制备的IL-COF-1作为固相萃取柱的填料,不同批次间目标物回收率的相对标准偏差(RSD)为5.2%~9.1%,表明IL-COF-1具有较好的制备重复性。

IL-COF-1的可重复使用性 对同一根固相萃取柱连续使用6次后,目标物回收率的RSD为2.5%~6.1%,表明IL-COF-1具有较好的可重复使用性。

IL-COF-1的稳定性 使用同一根IL-COF-1固相萃取柱在6天内(每天试验一次)获得的雌激素回收率为95.1%~107.4%, RSD值为6.2%~8.9%,表明其稳定性较好。

### 2.4 方法验证

分别吸取不同体积的单标储备溶液,加入到20 mL(含1 g蜂蜜)的水溶液中,得到含E_3_、E_2_和EE_2_ 1、10、25、50、100、250和500 ng/g以及E_1_和DES 0.1、0.5、1、10、25、50、100 ng/g的混合标准溶液,检测,绘制标准曲线。在最优条件下,如表2所示,该方法对E_3_、E_2_和EE_2_的线性范围为1~500 ng/g,对E_1_和DES的线性范围分别为0.1~100 ng/g,相关系数(*r*)为0.9934~0.9972。检出限(LOD, *S/N*=3)为0.01~0.30 ng/g,定量限(LOQ, *S/N*=10)为0.05~0.95 ng/g。

**表2:** SPE-HPLC-MS/MS方法分析性能

Compound	Linear range/(ng/g)	Correlation coefficient (r)	LOD/(ng/g)	LOQ/(ng/g)	RSDs/% (n=5)	
					Intra-day	Inter-day
E_3_	1-500	0.9934	0.25	0.80	4.4	6.7
E_2_	1-500	0.9955	0.25	0.80	6.6	7.9
EE_2_	1-500	0.9967	0.30	0.95	5.2	5.7
E_1_	0.1-100	0.9972	0.02	0.07	3.2	4.6
DES	0.1-100	0.9966	0.01	0.05	5.4	4.2

对含量为50 ng/g的5种雌激素进行重复性实验,日内精密度RSD为3.2%~6.6%,日间精密度RSD为4.2%~7.9%。

这些结果表明,基于IL-COF-1的SPE方法具有较高的灵敏度、良好的线性关系和较好的重复性。IL-COF-1材料作为SPE的吸附剂,可以用于蜂蜜样品中5种雌激素的检测。

### 2.5 方法应用

使用所开发的SPE-HPLC-MS/MS方法测定蜂蜜样品中的雌激素,2种蜂蜜样品中均未检测到5种雌激素。为了验证本方法的准确性,进行了加标回收试验,结果见表3和图4。3个加标水平(5、25、50 ng/g)下的回收率为80.14%~115.24%。以上结果表明,IL-COF-1作为吸附剂能很好地用于蜂蜜中雌激素的萃取。

**表3:** 蜂蜜样品中5种雌激素的加标回收率(*n*=5)

Estrogen	Added level/(ng/g)	Recoveries/%			
		Linden honey 1	Linden honey 2	Acacia honey 1	Acacia honey 2
E_3_	5	85.22±2.56	83.16±3.36	95.22±2.56	88.56±2.25
	25	88.96±4.25	86.97±4.14	88.52±3.65	89.67±4.21
	50	93.26±2.14	91.24±5.14	85.25±2.24	95.62±5.32
E_2_	5	102.52±4.57	85.66±4.21	99.58±3.33	89.64±2.28
	25	95.62±3.22	94.25±3.55	92.25±4.24	99.63±1.65
	50	107.32±3.54	97.45±4.14	87.42±1.15	87.58±4.15
EE_2_	5	86.45±4.78	97.36±5.21	87.66±4.41	92.35±2.27
	25	96.33±3.68	87.55±4.78	80.14±2.22	96.35±3.45
	50	85.67±4.58	99.25±4.57	86.35±4.25	90.24±2.27
E_1_	5	101.51±4.77	85.24±2.41	86.66±3.32	115.24±4.24
	25	83.24±3.98	88.44±2.57	88.57±2.26	87.36±3.56
	50	103.64±4.44	99.64±1.11	97.25±1.18	95.31±4.21
DES	5	86.53±3.89	87.21±2.25	97.58±2.26	88.65±4.21
	25	85.32±4.57	88.57±3.58	102.33±5.22	86.31±4.15
	50	95.51±4.67	96.80±4.22	103.52±3.31	99.65±3.67

**图 4:**
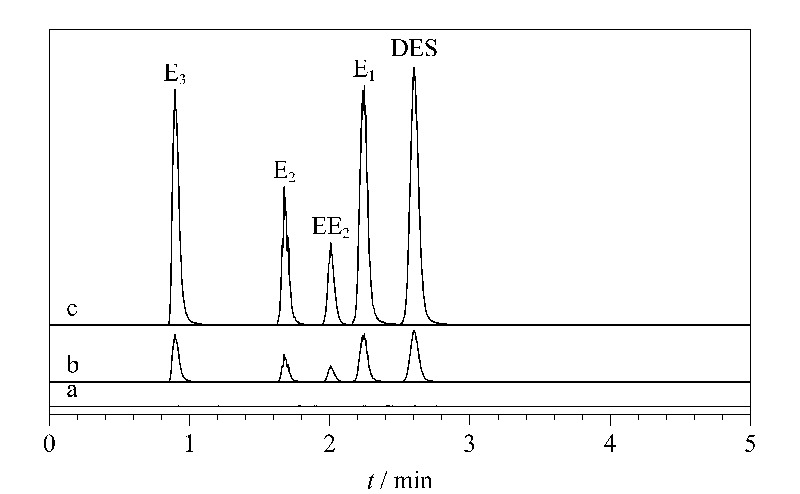
(a)空白蜂蜜样品、加标(b)5 ng/g和(c)25 ng/g 雌激素的蜂蜜样品的色谱图

在固相萃取过程中,IL-COF-1和雌激素之间可能存在如下相互作用:(i) IL-COF-1和雌激素中的芳香单元有利于形成*π-π*相互作用^[26]^; (ii)考虑到N和O等电负性原子的存在,雌激素和IL-COF-1之间也可能发生分子间O-H…N=C氢键相互作用^[27]^; (iii) IL-COF-1的高比表面积通过上述相互作用可增强雌激素与IL-COF-1的接触;(iv)孔径效应会阻止样品中大分子的通过,促进萃取过程中目标分析物的吸附和解吸。

## 3 结论

以IL-COF-1作为吸附剂,基于SPE技术和HPLC-MS/MS,建立了一种高效、灵敏的分析蜂蜜样品中痕量雌激素的新方法。方法学验证结果表明本方法具有较低的检出限和较宽的线性范围,稳定性和重复性良好。将本方法应用于实际蜂蜜样品分析,取得了满意的结果。IL-COF-1在雌激素的固相萃取中表现出优越的性能,需要进一步探索IL-COF-1在痕量分析中的应用。

## References

[1] Lafleur A D, Schug K A. Anal Chim Acta, 2011, 696(1/2): 6 21621029 10.1016/j.aca.2011.03.054

[2] Rao A, Douglas S C, Hall J M, et al. Cells, 2021, 10(6): 1439 34207527 10.3390/cells10061439 PMC8228950

[3] Mattiske D M, Pask A J. CRTOX, 2021, 2: 179 34345859 10.1016/j.crtox.2021.03.004 PMC8320613

[4] Mohajeri M, Bianconi V, Avila-Rodriguez M F, et al. Pharmacol Res, 2020, 156: 104765 32217147 10.1016/j.phrs.2020.104765

[5] Zhang Y J, Yu J, Xu J, et al. Modern Preventive Medicine, 2021, 48(6): 5

[6] Shi G Q, Li D, Lu X K, et al. Environment Chemistry, 2011, 30(1): 13

[7] Sun Y, Wu K B, Hu S S, et al. Microchim Acta, 2004, 142: 49

[8] Yang R, Liu J Y, Song D, et al. Microchim Acta, 2019, 186: 726 10.1007/s00604-019-3813-y 31655909

[9] Tang Z, Liu Z H, Wang H, et al. J Environ Manage, 2021, 292(10): 112804 34023789 10.1016/j.jenvman.2021.112804

[10] Alda M J L, Díaz-Cruz S, Petrovic M, et al. J Chromatogr A, 2011, 938: 145

[11] Xiong P, Gan N, Cui H, et al. Microchim Acta, 2014, 181: 453

[12] Zheng W, Wiles K N, Holm N, et al. J Am Chem Soc, 2014, 1171: 167

[13] Liu S Q. [MS Dissertation]. Shanghai: Shanghai Jiao Tong University, 2018

[14] Jana A, Mahugo-Santana C, Sosa-Ferrera Z, et al. Anal Chim Acta, 2011, 704: 33 21907019 10.1016/j.aca.2011.07.030

[15] Ma L, Ashworth D, Yates S R. J Pharmaceut Biomed, 2016, 131: 303 10.1016/j.jpba.2016.09.001 27616008

[16] Wu M, Miao E, Xu W, et al. Talanta, 2020, 219: 121272 32887162 10.1016/j.talanta.2020.121272

[17] Fan R T, Xiao H M, Cha D M, et al. Chinese Journal of Analytical Science, 2021, 37(3): 395

[18] Liu S Q, Liu R D, Wang W W, et al. Chinese Journal of Analysis Laboratory, 2019, 38(4): 470

[19] González A, Avivar J, Maya F, et al. Anal Bioanal Chem, 2017, 409: 225 27815608 10.1007/s00216-016-9988-8

[20] Wang X, Gu H L, Shen J, et al. Environmental Pollution & Control, 2018, 40(8): 890

[21] Glineur A, Beccaria M, Purcaro G. J Chromatogr A, 2021, 1652: 462359 34261020 10.1016/j.chroma.2021.462359

[22] Sun M, Li C Y, Feng J Q, et al. TrAC-Trends Anal Chem, 2022, 146: 116497

[23] Qian H L, Yang C X, Wang W L, et al. J Chromatogr A, 2018, 1542: 1 29496190 10.1016/j.chroma.2018.02.023

[24] Wang Z F, Zhang S N, Chen Y, et al. Chem Soc Rev, 2020, 49(3): 708 31993598 10.1039/c9cs00827f

[25] Rabbani M G, Sekizkardes A K, Kahveci Z, et al. Chem-Eur J, 2013, 19(10): 3324 23386421 10.1002/chem.201203753

[26] Xu G J, Zhang B B, Wang X L, et al. Microchim Acta, 2019, 186(1): 26

[27] Sharath K, Digambar B S, Manas K P. Angew Chem Int Edit, 2013, 52(49): 13052

